# The importance of social support in the associations between psychological distress and somatic health problems and socio-economic factors among older adults living at home: a cross sectional study

**DOI:** 10.1186/1471-2318-12-27

**Published:** 2012-06-08

**Authors:** Hege Bøen, Odd Steffen Dalgard, Espen Bjertness

**Affiliations:** 1 Division of Mental Health, Department of Surveillance and Prevention, Norwegian Institute of Public Health, Oslo, Norway; 2 Faculty of Medicine, UiO, Department of Community Medicine, Institute of Health and Society, Oslo, Norway

**Keywords:** Older adults, Social support, Psychological distress, Somatic health, Social inequality

## Abstract

**Background:**

Little is known of the importance of social support in the associations between psychological distress and somatic health problems and socio-economic factors among older adults living at home. The objectives of the present study were to investigate the associations of social support, somatic health problems and socio-economic factors with psychological distress. We also examined changes in the association of somatic health problems and socio-economic factors with psychological distress after adjusting for social support.

**Methods:**

A random sample of 4,000 persons aged 65 years or more living at home in Oslo was drawn. Questionnaires were sent by post, and the total response was 2,387 (64%). Psychological distress was assessed using Hopkins Symptom Checklist (HSCL-10) and social support with the Oslo-3 Social Support Scale (OSS-3). A principal component analysis (PCA) included all items of social support and psychological distress. Partial correlations were used, while associations were studied by logistic regression.

**Results:**

After adjusting for socio-demographics and somatic health problems, we reported a statistically significant association between psychological distress and social support: “Number of close friends”, OR 0.61; 95% CI 0.47-0.80; “Concern and interest”, OR 0.68; 95% CI 0.55-0.84. A strong association between lack of social support and psychological distress, irrespective of variables adjusted for, indicated a direct effect. The associations between psychological distress and physical impairments were somewhat reduced when adjusted for social support, particularly for hearing, whereas the associations between somatic diagnoses and psychological distress were more or less eliminated. Income was found to be an independent determinant for psychological distress.

**Conclusions:**

Lack of social support and somatic health problems were associated with psychological distress in elders. Social support acted as a mediator, implying that the negative effect of somatic health problems, especially hearing, on psychological distress was mediated by low social support. We hypothesize that physical impairments reduced social support, thereby increasing psychological distress to a greater extent than the selected diagnoses. The combination of poor social support, poor somatic health and economic problems may represent a vulnerable situation with respect to the mental health of older persons. Free interventions that highlight social support should be considered in mental health promotion.

## Background

Little is known about how associations between psychological distress and somatic health problems are mediated by social support among the elderly. Several studies have documented associations between psychological distress and poor somatic health, low socio-economic level and weak social support. It is unclear, however, whether good social support can improve psychological distress despite poor somatic health and a low level of socio-economy. In this cross-sectional study, we investigate how associations between psychological distress, somatic health problems (diagnoses and physical impairments) and socio-economic factors are mediated by social support.

Different studies point to both the impact of physical health and social support on the mental health of older persons [1-3]. Late-life depression is perhaps the most frequent cause of emotional suffering, and is also found to be a risk for poor self-rated health over time [4]. Health also shows a strong social gradient. The prevalence of psychological distress increased by decreasing social status [5].

### Social support and mental health

Elders could be faced with greater losses in the context of fewer social resources and a lower adequacy of social support, both in subjectively perceived support and frequency of contacts. Social relationships, ranging from social isolation to social support, have long been implicated as being at risk for depression [6]. It is generally agreed that social support plays a beneficial role in the maintenance of mental health and psychological well-being (and reduces the risk of depression). There are two alternative causal models which are common in explaining how social support affects psychological distress, the direct effect model and the indirect (buffer) effect model [7]. The direct effect implies that social relationships have a beneficial effect on health, regardless of life situation, whereas the stress-buffering effect implies that social relationships only have a beneficial effect for persons exposed to stressors, such as negative life events and hardships over time. In this instance, social support is thought to buffer the effects of stress by enhancing personal coping abilities such as self-esteem and self efficacy. Through a strengthening of the coping mechanism, the negative emotional reaction to a stressful event will either be reduced, or the physiological responses on health *via* the immune system will be dampened [8-11].

### Somatic health and mental health

Somatic health problems carry a high risk of anxiety disorder and depression, with depression producing the greatest decrements in health compared with other chronic diseases [12]. Disability and depressive symptoms are mutually reinforcing over time against a potential downward trend for disabled elderly adults, and the effect of disability on depression has been shown to be faster and stronger than the effect of depression on disability [13].

### Social support and somatic health

Studies show that poor social support increases both the risk of somatic health problems and mortality among elders [14,15], although there are also studies which demonstrate that somatic health problems have a negative effect on social support [16]. The negative effect of somatic health problems on social support implies that social support may be a mediator in the relationship between somatic disorders and psychological distress, and not only a moderator or buffer as mentioned above. Because somatic health problems tend to reduce social support, which is a risk factor for mental health problems, somatic health problems increase the risk of mental health problems, though few studies have looked into this pathway.

### Conceptual model of the hypothesized relationships among variables

**Figure 1:**
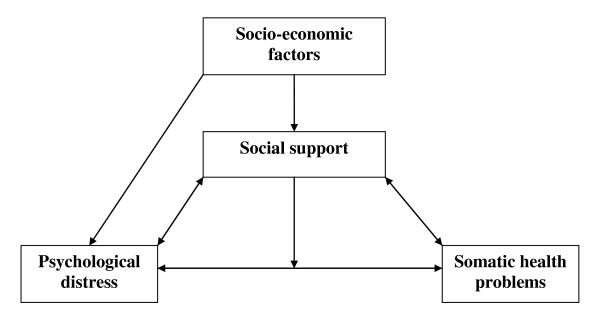
Conceptual model of the relationships between social support, psychological distress, somatic health problems and socio-economic factors.

The relationships among the variables of social support, psychological distress, somatic health problems and socio-economic factors are illustrated in Figure1

Between psychological distress and social support, we expect a direct effect that goes both ways. Psychological distress results in decreased social support, while decreased social support increases psychological distress. Furthermore, we expect social support to act as a moderator on the relationship between psychological distress and somatic health problems, which means that the strength of the relationship between psychological distress and somatic health problems is dependent on social support. We also expect that social support will act as a mediator between psychological distress and somatic health problems since psychological distress affects social support, and we expect social support to affect somatic health problems. Additionally, a low socio-economic status is associated with psychological distress and low social support [17,18]. We expect that such associations will also be found in this material, as these aspects seen together provide the rationale for this study.

The objective of the present study of persons aged 65 and above living at home was to investigate the association between psychological distress and social support. We wanted to explore whether social support mediates or moderates the association of psychological distress with somatic health problems. Moreover, we investigated the association between psychological distress, somatic health problems and socio-economic factors. Finally, we examined changes in the association between somatic and socio-economic factors after adjusting for social support.

We hypothesized that:

1. Social support has a direct effect on psychological distress;

2. Social support acts as a mediator between psychological distress and somatic health problems such as diagnoses and physical impairments;

3. Social support acts as a moderator between psychological distress and somatic health problems such as diagnoses and physical impairments.

## Methods

### Sample design and data collection

The data in this cross-sectional study was collected in 2006, using the Norwegian Population Register from one eastern (Østensjø) and one western (Ullern) district of Oslo, and the median age was 77 years in Østensjø and 76 years in Ullern. The Norwegian National Population Register is a public register with details of all registered residents living in Norway, and is administered by the Norwegian Tax Administration.

A random sample of 4,000 persons aged 65 years or more living at home, with 2,000 from each district, was drawn. Of the random sample, 111 persons were residents of institutions and were therefore excluded from the sample. Letters of information and questionnaires were sent to 3,889 persons by post, and a reminder was sent two weeks later, which resulted in a total response of 2,387 (64%) participants. The questionnaires were scanned and quality controlled, and a total of 2,387 (64%) out of 3,889 questionnaires were included in the analysis.

Table1 shows the distribution of the study population contrasted with the total number of citizens over 65 years of age in Ullern and Østensjø.

**Table 1:** Study population stratified by gender and age within city districts (n = 2387)

		Total number*	**Invitees**	**Respondents**
		n = 17,525	n = 3,889	n = 2,387
	Age	n (%)	n (%)	n (%)
ULLERN				
	65-69	889 (24)	237 (31)	112 (22)
Men	70-79	1,673 (45)	302 (39)	226 (44)
	80+	1,138 (31)	229 (30)	177 (34)
	65-69	1,018 (18)	258 (22)	142 (20)
Women	70-79	2,393 (42)	436 (38)	310 (43)
	80+	2,320 (40)	466 (40)	267 (37)
ØSTENSJØ				
	65-69	536 (18)	159 (22)	75 (17)
Men	70-79	1,555 (52)	347 (48)	233 (53)
	80+	911 (30)	217 (30)	135 (30)
	65-69	823 (16)	233 (19)	125 (18)
Women	70-79	2,457 (48)	580 (47)	354 (50)
	80+	1,812 (36)	425 (34)	231 (32)
Total				
Men		6,702 (38)	1,491 (38)	958 (40)
Women		10,823 (62)	2,398 (62)	1,429 (60)

* Oslo statistics for age group 65 and older in the city districts of Ullern and Østensjø, collected by Statistics Norway.

### Variables

Data were collected by self-report using a questionnaire with fixed-answer alternatives for 40 questions. Not all of the respondents answered every question, so consequently the numbers included in the analysis vary slightly. For the questions on income and marital status, 6% and 1% of participants, respectively, did not answer, while the percentage of other questions left unanswered was less than 1%.

### Psychological distress

The indicator of psychological distress, was measured using the Hopkins Symptom Checklist (10 questions HSCL-10), which is the short form of a battery of 25 questions (HSCL-25) measuring the symptoms of anxiety and depression [19]. A score of 1.85 or higher indicates symptoms of anxiety and depression that interfere with daily living, but do not necessarily require treatment [20]. The HSCL-10 is recommended for screening purposes because this scale represents the best compromise between economy and accuracy in identifying “distressed” and “non-distressed” groups in the general population [21].

### Social support

Social support was measured using the Oslo-3 Social Support Scale (OSS-3) with three questions [22]. The response categories were assessed independently for each of the three questions, and a sum score was created by summarizing the raw scores. The Oslo-3 scale has been used in several studies, thus confirming its feasibility and predictive validity with respect to psychological distress [22-24]. In this study, the scale is used as both a sum score and an item-by-item scale. To state the prevalence of social support, we used the sum score scale ranging from 3–14, which was then operationalized into three broad categories: “poor support” 3–8, “moderate support” 9–11 and “strong support” 12–14 (Table2). To test the correlations between physical impairments and diagnoses, the scale was used both item-by-item and by sum score (Table3). In a logistic regression (Table4), we used the scale item-by-item to explore the contribution and changes of the three items (Oslo1,2,3) when adjusted for all variables.

**Table 2:** Distribution of socio-demographics, diagnoses, physical impairments, social support and psychological distress by gender

	**n (%)**	**n (%)**	**n (%)**
	Women n = 1,429	Men n = 958	Total n = 2,387
**Socio-demographics**			
Age group			
65-69	268 (19)	187 (20)	455 (19)
70-79	667 (46)	459 (48)	1,126 (47)
80+	501 (35)	312 (33)	813 (34)
Education			
Primary, 9 yrs	800 (56)	373 (39)	1,173 (50)
Secondary, 12 yrs	234 (17)	122 (13)	356 (15)
College/Univ > 12 yrs	388 (27)	454 (48)	842 (36)
Income in thousands*			
150’	591 (45)	90 (10)	681 (30)
150-200’	273 (21)	167 (18)	440 (20)
200-300’	293 (22)	241 (26)	534 (24)
300’	172 (13)	434 (47)	606 (27)
Marital status			
Married/cohabiting	633 (45)	742 (80)	1,375(58)
Single	791 (56)	211 (22)	1,002(42)
District of town			
Ullern	719 (50)	515 (54)	1,234 (52)
Østensjø	710 (50)	443 (46)	1,153 (48)
**Diagnoses** (dichotomized)			
Diabetes	87 (6)	94 (10)	181 (8)
Chronic lung disease	96 (7)	44 (5)	140 (6)
Osteoporosis	260 (18)	22 (2)	282 (12)
Musculoskeletal ailment	607 (42)	216 (23)	823 (34)
Cardiac infarction	80 (6)	126 (13)	206 (9)
Angina	99 (7)	104 (11)	203 (9)
Stroke	118 (8)	99 (10)	217 (9)
Cancer	216 (15)	125 (13)	341 (14)
**Physical impairment** (dichotomized)			
Balance	617 (44)	322 (34)	939 (40)
Vision	450 (32)	228 (24)	678 (29)
Hearing	513 (36)	435 (46)	948 (41)
Urine leak	444 (32)	334 (35)	778 (33)
Memory	505 (36)	362 (38)	867 (37)
**Social support (three items)** Number of friends to count on			
None	51 (4)	17 (2)	68 (3)
1-2	431 (30)	245 (26)	676 (29)
3-5	557 (39)	403 (42)	960 (41)
5+	381 (27)	288 (30)	669 (28)
Concern from others			
A lot	396 (30)	261 (29)	657 (29)
Some	583 (44)	423 (46)	1,006 (45)
Uncertain	262 (20)	164 (18)	426 (19)
Little	59 (5)	45 (5)	104 (5)
None	23 (2)	20 (2)	43 (2)
Practical help			
Very easy	118 (9)	93 (10)	211 (9)
Easy	248 (18)	192 (21)	440 (19)
Possible	551 (40)	404 (44)	955 (42)
Difficult	271 (20)	161 (18)	432 (19)
Very difficult	190 (14)	70 (8)	260 (11)
**Social support (sum score)**			
Poor support	357 (28)	193 (22)	550 (25)
Moderate support	656 (51)	469 (53)	1,125 (52)
Strong support	275 (21)	227 (26)	502 (23)
**HSCL-10 > 1.85**			
Total	136 (12)	36 (4)	172 (8)
65-69	20 (8)	6 (3)	
70-79	56 (10)	16 (4)	
80+	60 (16)	14 (5)	

* Norwegian kroner (NOK).

**Table 3:** Partial correlations between the categories of social support and somatic health problems adjusted for gender and age (n = 2,387)

**Variables**	**Number of friends to count on**	**Concern from others**	**Practical help Total social support**
**Physical impairments** Balance	-.192**	-.163**	-.184**
Vision	-.155**	-.141**	-.151**
Hearing	-.106**	-.104**	-.098**
Urine leak	-.124**	-.139**	-.127**
Memory	-.141**	-.170**	-.104**
Impairments	-.234**	-.234**	-.212**
sum			-.299**
**Diagnoses** Diabetes	-.008	-.071**	-.012
Chronic lung disease	-.069**	-.039	-.058**
Osteoporosis	-.078**	-.038	-.089**
Musculoskeletal ailments	-.075**	-.059**	-.072**
Cardiac infarction	-.039	-.067**	-.013
Angina	-.075**	-.057**	-.070**
Stroke	-.052*	-.070**	-.067**
Cancer	-.033	-.042*	-.062*
Diagnoses	-.139**	-.139**	-.145**
sum			-.194**

p-values; *p < 0.05; **p < 0.01.

**Table 4:** Associations ORs and 95% CI between dependent variable: psychological distress (HSCL-10 ≥1.85), socio-demographics, somatic health-problems and social support (OSS-3), (n = 2,387)

I**ndependent variables**	**Model 1 Each predictor adjusted for age and gender**	**Model 2a and b Adjusted for age, gender and social support**	**Model 3a and b Adjusted for age, gender, social support and socio-demographics**	**Model 4 Adjusted for all variables**
**Social support**				
Number of close friends	0.44*** (0.36 – 0,54)			0.61*** (0.47 – 0.80)
Concern and interest	0.52*** (0.44 – 0.61)			0.68*** (0.55 – 0.84)
Practical help from neighbours	0.74*** (0.64 – 0.86)			1.13 (0.93 – 1.37)
**Demography**				
Education				
Primary, 9 yrs (ref)	1.00			1.00
Secondary, 12 yrs	0.55* (0.33 – 0.90)			0.75 (0.39 – 1.43)
College/Univ > 12 yrs	0.60** (0.41 – 0.88)			1.04 (0.61 – 1.75)
Income in thousands				
150’ (ref)	1.00			1.00
150-200’	0.51** (0.33 – 0.81)			0.53* (0.31 – 0.91)
200-300’	0.42*** (0.27 – 0.65)			0.54* (0.31 – 0.94)
300+	0.21*** (0.12 – 0.39)			0.31* (0.19 – 0.83)
Marital status				
Married/cohabiting (ref)	1.00			1.00
Single	1.48* (1.05 – 2.08)			1.37 (0.90 – 2.08)
District of town				
Ullern (ref)	1.00			1.00
Østensjø	1.71** (1.24 – 2.36)			1.10 (0.71 – 1.72)
**Somatic health problems, 2a,b and 3a,b**				
**Diagnoses 2a and 3a**				
Diabetes	1.45 (0.83 – 2.53)	1.37 (0.76 – 2.47)	1.37 (0.73 – 2.58)	0.86 (0.41 – 1.81)
Chronic lung disease	2.04** (1.21 – 3.46)	1.68 (0.95 – 2.98)	1.55 (0.85 – 2.81)	1.25 (0.67 – 2.35)
Osteoporosis	2.52*** (1.71 – 3.71)	2.28*** (1.51 – 3.46)	2.38*** (1.54 – 3.68)	1.59 (0.98 – 2.56)
Musculoskeletal ailments	2.19*** (1.59 – 3.03)	1.90*** (1.35 – 2.69)	1.77** (1.23 – 2.53)	1.50* (1.01 – 2.22)
Cardiac infarction	2.09** (1.29 – 3.37)	1.85* (1.10 – 3.10)	1.78* (1.04 – 3.05)	1.70 (0.94 – 3.08)
Angina	2.10** (1.32 – 3.34)	1.67* (1.01 – 2.77)	1.72* (1.02 – 2.90)	1.25 (0.69 – 2.26)
Stroke	2.09** (1.34 – 3.28)	1.75* (1.07 – 2.85)	1.44 (0.86 – 2.40)	0.87 (0.50 – 1.51)
Cancer	1.01 (0.66 – 1.55)	0.85 (0.54 – 1.36)	0.79 (0.48 – 1.29)	0.87 (0.51 – 1.47)
Diagnoses, sum	1.70*** (1.48 – 1.96)	1.53*** (1.32 – 1.78)	1.50*** (1.27 – 1.77)	1.29** (1.08 – 1.54)
**Physical impairment, 2b and 3b**				
Balance	6.58*** (4.41 – 9.83)	5.16*** (3.41 – 7.81)	5.53*** (2.97 – 6.91)	2.66*** (1.67 – 4.22)
Vision	4.06*** (2.92 – 5.65)	3.31*** (2.33 – 4.70)	3.07*** (2.12 – 4.44)	2.21*** (1.48 – 3.31)
Hearing	1.59** (1.14 – 2.21)	0.79 (0.96 – 1.94)	1.30 (0.90 – 1.87)	0.87 (0.58 – 1.30)
Urine leak	3.42*** (2.45 – 4.78)	3.02*** (2.11 – 4.31)	3.13*** (2.16 – 4.54)	2.02*** (1.43 – 3.20)
Memory	3.63*** (2.57 – 5.11)	3.32*** (2.31 – 4.78)	3.06*** (2.10 – 4.47)	1.99*** (1.31 – 3.00)
Impairments, sum	2.10*** (1.84 – 2.38)	1.98*** (1.72 – 2.27)	1.90*** (1.65 – 2.20)	1.84*** (1.59 – 2.13)

Model 1: Separate logistic regression analyses with each predictor one-by-one, controlled for age and gender.

Models 2a and b: Logistic regression analyses controlled for age, gender and social support, with separate analyses for diagnoses and physical impairments.

In Model 2a, we have treated all diagnoses as one group without physical impairments.

In Model 2b, we have treated all physical impairments as one group without diagnoses.

Models 3a and b: Logistic regression analyses controlled for age, gender, social support and other socio-demographic factors, with separate analyses for diagnoses and physical impairments.

In Model 3a, we have treated all diagnoses as one group without physical impairments.

In Model 3b, we have treated all physical impairments as one group without diagnoses.

Model 4: Logistic regression analyses controlled for all variables introduced simultaneously, including age, gender, social support, socio-demographic factors, diagnoses and physical impairments.

*p < 0.05; **p < 0.01; ***p < 0.001.

Oslo 1: How many people are you so close to that you can count on them if you have great personal problems? (none (1), 1–2 (2), 3–5 (3), 5+ (4))

Oslo 2: How much interest and concern do people show in what you do? (a lot (5), some (4), uncertain (3), little (2), none (1))

Oslo 3: How easy is it to get practical help from neighbours if you should need it? (very easy (5), easy (4), possible (3), difficult (2), very difficult (1))

The way that social support and psychological distress are defined in the present study raises the question of whether we are dealing with distinct constructs other than psychological distress. To explore the underlying structure and proximality of the HSCL-10 and OSS-3 scales, a principal component analysis (PCA) was used. A varimax rotation technique with Kaiser Normalization was used as a component extraction method. The correlations between each item of social support and psychological distress have been estimated, and a PCA has been carried out including, all the items of social support and psychological distress. A correlation analysis exhibits moderate correlations between psychological distress and the three social support items (HSCL-10 and (1) number of friends to count on, -.264**, (2) concern from others, -.271**, (3) practical help, -182**), which indicates that we are dealing with different constructs (not shown in the table). This is confirmed in the principal component analysis, in which the items of psychological distress and social support are clearly loading on two different components. Of the total variance, 39% is explained by Component 1 and 15% by Component 2.

### Somatic health problems

Somatic health problems were measured by dichotomized questions (yes/no) about the presence of eight frequently occurring *diagnoses*: diabetes, chronic lung disease, osteoporosis, musculoskeletal ailments, coronary infarct, angina, stroke and cancer. The question to be answered was: “Do you have or have you had some of the listed diagnoses?” The *physical impairments* covered were those of balance, hearing, vision, continence and memory, all of which are also common in older years. Dichotomized questions (yes/no) about the present status were asked about the physical impairments.

### Socio-economic status

Socio-economic status was measured by educational level and income, with the educational level ranging from nine years of primary school, 12 years of secondary school and more than 12 years of college/university. The income was given in thousands (Norwegian kroner (NOK)), including 150’, 150-200’, 200-300’, 300’ or more.

### Statistical methods and analyses

Frequencies and cross tabulations gave the distribution of socio-demographic variables, diagnoses, somatic health-problems, social support and psychological distress.

Partial correlations between social support and somatic health problems (measured by physical impairments and diagnoses) adjusted for the effect of gender and age were performed (Table3). To find out whether there were differences in the strength of the correlations, linear regression analyses were used to investigate possible significant differences between the sum score of the impairments and the sum score of the diagnoses with respect to social support, which were adjusted for gender and age (not shown in table).

A logistic regression was performed to assess the associations between independent variables (social support, demographic variables, diagnoses and physical impairments) and psychological distress (see Table4, Models 1–4). According to our analytic strategy, each predictor variable in Model 1 was adjusted for gender and age one-by-one; hence, Model 1 consists of a series of separate regression analyses. In Model 2, the associations between diagnoses, physical impairments and psychological distress were additionally adjusted for the three categories of social support, while we also adjusted for socio-demographic variables in Model 3. In the final model, Model 4, we adjusted for diagnoses and physical impairments as well (*i.e.* all variables). The hypothesis of a *direct effect* of social support on psychological distress was conclusively tested in Model 4, Table4. The hypothesis of a *mediator effect* of social support on diagnoses, physical impairments and psychological distress was tested in Models 2 and 3, Table4, with an adjustment for social support and demographic factors.

Lastly, we carried out a multiplicative interaction analysis to see whether social support had a “buffer” or *moderator function,* and binary logistic regression analyses with interaction effects were performed. The analyses were conducted between each socio-demographic variable, each somatic health variable and the social support sum score, which was further operationalized into poor, moderate and strong support with respect to psychological distress and an adjustment for age and gender.

The level of significance was set to p ≤ 0.05, or confidence interval, CI = 95%. SPSS (Statistical Package for the Social Sciences) version 17 was used in the data analysis.

## Results

Table2 gives the distribution of demographic characteristics, diagnoses, physical impairments, psychological distress and social support by gender and for the total sample:

The percentage of physical impairments in each of the indices was 29-41%. Hearing impairment was the most prevalent at 41%, whereas musculoskeletal ailments proved to be the most common diagnosis at 34%. For women, we reported three times as high a prevalence of psychological distress than for men. The sample reflects well-known gender differences in psychological distress, although psychological distress increased with age for both women and men, and its prevalence was 8.4%. Poor social support (score 3–8) was most frequent in women, with a total of 25%.

The results of the correlation analyses between social support (three categories), physical impairments and diagnoses are given in Table3:

There were significant negative correlations between social support and almost all of the physical impairments and diagnoses, r = −.042 to -.192, adjusted for age and gender. Correlations showed generally higher correlation coefficients between social support and physical impairments than for social support with diagnoses. Total social support showed higher correlation with impairments sum score r = −.299, than with diagnoses sum score r = −.194. The strength of the correlations in linear regression did not prove statistically different for the diagnoses and impairments, but a tendency was shown. The confidence intervals (CIs) overlapped each other, (−.313, -.130) for diagnoses and

(−.390, -.259) for impairments. Social support decreased for all three categories of social support when physical impairments and diagnoses were present.

The associations between HSCL-10 cut-off ≥1.85 and social support, demographic characteristics, diagnoses and physical impairments adjusted for gender and age, are presented in Models 1–4, Table4.

Most independent variables were significantly associated with HSCL-10. A high level of education, income and good social support were significantly associated with a low psychological distress, while being single, living in the eastern district (Østensjø) and having physical impairments and diagnoses, except for diabetes and cancer, displayed significantly higher odds for psychological distress (Model 1, adjusted for gender and age one-by-one).

The observed associations, adjusted for age and separately analysed by gender, showed no substantial differences in OR between women and men (not shown in table).

After an additional adjustment for social support (Model 2), hearing lost its position as a significant independent predictor for psychological distress. Nevertheless, the rest of the physical impairments still demonstrated a strong significant association with psychological distress, although the ORs were reduced in comparison to Model 1. Separate analyses with an introduction of the social support variables one-by-one revealed that all three contributed to the reduction of ORs.

After an additional adjustment for demographic variables (Model 3), the estimates changed marginally, with only stroke no longer proving significant.

When adjusted for all variables, Model 4, “practical help from neighbours” was no longer a significant determinant of psychological distress. The “number of close friends” and “concern and interest from others” remained consistent. Education, marital status and living in the eastern part of Oslo exhibited no significant association with psychological distress in Model 4, as contrasted to the analyses in Model 1. Among the demographic characteristics, income was still an independent determinant with some reduction in OR. The association between physical impairments and psychological distress was somewhat reduced when adjusted for all variables but was still significant, whereas the associations between diagnoses and psychological distress were more or less eliminated. Also, the sum score diagnoses and sum score impairments remained consistent as independent predictors of psychological distress, although somewhat reduced when contrasted to Model 1.

To investigate whether the associations between HSCL-10 and somatic- and socio-demographic factors varied by different levels of social support, interaction tests between social support and demographics, the five impairments and the eight diagnoses with respect to psychological distress were carried out adjusted for gender and age, though none of these tests proved significant.

## Discussion

The main findings of the present study were that a significant and consistent association was found between social support and psychological distress regardless of the variables adjusted for (direct effect). The associations between psychological distress and physical impairments were somewhat reduced when adjusted for social support, particularly for hearing (mediator effect), whereas the associations between somatic diagnoses and psychological distress were more or less eliminated. Income also maintained its position as an independent determinant for psychological distress when adjusted for all variables.

### Direct effect of social support on psychological distress

In relation to “number of close friends” and “concern and interest from others”, social support was significantly independently associated with psychological distress through the multivariate analyses, whereas “practical help from neighbours” lost its significance. A likely explanation for this could be that the three factors of social support were interrelated, and that the neighbour factor was explained through the other two. The finding that social support is important for the mental health of the elderly is in accordance with the finding of other studies [25,26], and the fact that you have someone you can trust to turn to when experiencing great personal problems, not to mention the concern shown by other people towards what you are doing, is important in diminishing psychological distress. Family is known to be an important source of social contact in older years, and though we know less about the importance of friendship, friends seem to be of great importance in the present study. The reason for this may be because friends represent a source of identity with regard to usually being the same gender and age, sharing experiences and staying close through hardships such as the death of a spouse or other important life events. It seems that cultural norms for close ties held by older people differ little from the rest of one’s lifespan, as norms of trust, commitment and respect are important to them as well [27].

### Social support as a mediator between psychological distress and somatic health problems

The associations between somatic disorders, psychological distress, and to a lesser extent physical impairments, were somewhat reduced when adjusting for social support, thereby helping the hypothesis of a mediator function gain some support. It seems as if the negative effect of somatic disorders on psychological distress is explained to some degree by somatic disorders that lead to reduced social support. It is interesting to note that the association between hearing loss and psychological distress was relatively strongly reduced when adjusting for social support. It is likely that an impairment of this type in particular, which is one of the most common chronic somatic disorders in the elderly, leads to reduced social contact and support, and therefore to increased psychological distress. This study showed hearing impairment to be the most prevalent of the somatic disorders by 41%, while the negative effect of hearing loss on social contacts is in agreement with other studies [28,29]. The burden of hearing impairment increased due to communication problems and a lack of social support, with social isolation and loneliness as the consequences, which further led to increased psychological distress. Hence, this study demonstrated itself to be a good example of the role of social support as a mediator between hearing impairment and psychological distress.

The finding that somatic health problems are strongly associated with psychological distress is in accordance with the findings of a number of other studies [30-32], and it is interesting to note that physical impairments seem more strongly associated with psychological distress than diagnoses, and that the associations between diagnoses and psychological distress were more or less eliminated when adjusted for other variables, including physical impairments. This may indicate that the negative effect of diagnoses on mental health is partially mediated by impairments, and that it is the impairments that are the ones most strongly interfering with daily life.

All physical impairments and diagnoses, with the exception of cancer, were negatively correlated with each category of social support, though the correlations seemed stronger between physical impairment and social support than between diagnoses and social support. Given the cross-sectional nature of the data, it is difficult to decide what is cause and effect in the relationship between social support and somatic health, as both directions of causality are possible [14,15,33]. It is not likely however, that lack of social support should be a cause of impairments for hearing, urine leaks, vision and balance, although it could be both the cause of memory impairment and the result of it. Some studies show that good cognitive functioning is associated with social integration and support [34], as well as the prevention of dementia [35,36].

The issue of co-morbidity is important with respect to the number of diagnoses and the number of impairments, which raises the question of why persons with impairments receive less social support than persons with diagnoses. One explanation for this can lie in the fact that the practical and social consequences of impairments and diagnoses differ in daily life. Problems with balance, hearing, vision, urine function and memory may cause poor communication, information decrease and mobility problems, and are socially stigmatizing and connected with aging and mental decline. For instance, associations between recent vision impairment and changes in social life have been shown in recent studies, while older people with recent vision impairment reported being lonelier and having a reduced social interaction and declined mood, as shown by others in relation to vision impairment [37,38]. The daily consequences of the medical diagnoses in question are of course severe, visible and troublesome as well, though not to the same degree in connection to age decline since these diagnoses also affect younger people and are connected to the patient’s role, which might generate more social and medical benefits than impairments.

There were no interactions between somatic health problems and social support with respect to psychological distress, thus the hypothesis of moderator function was not supported. This finding was not in agreement with other findings [30-32,39], which may be because the measure of social support used is in the present study was of a more general nature, and not sensitive to the actual experience of support linked to somatic health problems or other specific events.

### Income as an independent determinant for psychological distress

Both mental health and somatic health show a social gradient, with the prevalence of illness increasing by a decreasing socio-economic status [5,17]. A low socio-economic status is also associated with low social support, and a lack of support explains some of the social gradient in mental health. Especially in older adults, a deterioration in financial status is known to be a stressful event, and those who are economically disadvantaged are more likely to experience persistent depressive symptoms [18]. Therefore, socio-economic status was taken into consideration as a possible confounder when analysing the relationship between social support and psychological distress, with income maintaining its position as an independent protective factor for psychological distress, also when adjusted for health and social support in the final multivariate model. Associations between education, marital status, district of town and psychological distress become non-significant in the multivariate analyses when adjusting for all items. This confirms the assumption that financial strain is a source of psychological distress for many older adults [40-42], and that the challenge of social inequalities in health is also present in the elder age groups. In this material, the odds ratio is 3 for economic problems for experiencing high levels of psychological distress among those with poor somatic health (level of significance 1%). In planning structural initiatives targeting psychological distress as a public health issue, it is important to avoid those in poorer socio-economic conditions being less involved than those who are better socially positioned. This implies that such activities should be free of charge.

### Strengths and limitations

The study respondents seemed to be representative of both the total number and of the invitees concerning age and gender in both districts, except for a small underrepresentation among men in the youngest age group in Ullern. The question on income did not specify whether gross, net or adjusted household income was asked for, which leaves room for different interpretations, although we assume that most of the respondents reported their gross income.

Several previous studies have investigated samples from primary health care and hospital settings. This study investigated a random sample of home living, and we had pre-formulated hypotheses. The response rate was high, and the sample seemed fairly representative of the target population with respect to age, gender and place of living. However, missing one-third of the sample is a concern that could possibly lead to selection bias, as it is possible that we have reached the fittest in this survey. However, the time span between a fully upgraded available list of addresses and invitations sent was about six months. During that time, some of the potential respondents with extensive somatic health problems and/or suffering from a lack of social contact may have moved to institutions or died. Hence, the associations are at least not overestimated. Validations ofHSCL-10, the Social Support Scale and the OSS-3 indicate that they are regarded as valid and reliable instruments. It is a possible weakness that the information on somatic health came from self-reports, and not from medical examinations. Even so, it is regarded as easier to admit rather embarrassing health problems in a postal survey than in a face-to-face interview. Understating somatic health problems and reporting too much loneliness will only weaken the associations, whereas the approach that tests whether social support serves more as a moderator or mediator in the relationship between physical and mental health is a strength of the study.

It is an important limitation that the study is based on a cross-sectional design, which does not allow for drawing conclusions on causality. In this study, there was a potential bias in the recall of social support among distressed individuals, with such individuals being more inclined to describe their social support in more negative terms than others. Such a dependency in the data may therefore lead to false associations [43]. Another possibility for reversed causality could be that social isolation and a lack of social support are consequences of mental health problems. Certain personality traits such as introversion are associated with both a lack of social network participation and the occurrence of depressive symptoms [44]. The principal component analysis of psychological stress and social support confirmed that the two measures clearly loaded on two different components, which indicates that although psychological distress and social support correlate, there is no element of symptomatology or trait vulnerability in this correlation. The two measures operate as two different constructs.

### Summary of social support and health

Hearing loss and other common losses of vital functions lead to isolation and a lack of social relations. The impairments become an additional load factor that increases loneliness as in a vicious circle, and a lack of social support and impairments increased psychological stress in older persons. In this study, 25% experienced poor social support (Table2), and it seems that social support is as equally important as physical health in preventing psychological distress, thereby making it a natural target for prevention and health promotion. Impairments reduce social support to a larger extent than diagnoses. This is a serious public health concern since impairments are quite common among older persons, with between 29 to 41% in this study reporting physical impairments and between 8 and 34% reporting diagnoses.

### Practical implications

It is important that a lack of social support and somatic health problems are addressed in mental health promotion among older people since they are both important risk factors for psychological distress. In combination with an awareness of possible somatic health problems, an increased focus on initiating and implementing interventions that highlight social support, especially in relation to hearing impairments, seems to be a good strategy. A senior centre is a valuable service provision in this context, serving both fit and less functional pensioners free of charge. The goal of a senior centre is to maintain physical and psychological activity and functional health, in addition to strengthening social support [45]. Further research needs to address different health and social service trials aiming to promote functional and mental health by social support.

## Conclusions

This study revealed that a lack of social support and somatic health problems were associated with psychological distress in elders, as social support seemed to have a direct effect on psychological distress. There was also some support for the mediator hypothesis, implying that the negative effect of somatic health problems for hearing in particular on psychological distress was, to some extent, mediated by weakened social support. Physical impairments reduced social support to a larger degree than diagnoses, though no support to the moderator or “buffer” hypothesis was found, while income was found to be an independent determinant for psychological distress. The combination of weak social support, poor somatic health and economic problems may represent an extremely vulnerable situation with respect to the mental health of older persons. Free of charge interventions that highlight social support should be considered in mental health promotion.

## Ethical approval

The study was approved by the Norwegian Data Inspectorate and recommended by the Regional Committee for Medical Research Ethics, Southern Norway in September 2006.

## Competing interests

The author(s) declare that they have no competing interests.

## Authors’ contributions

HB was responsible for the data collection. HB, OSD and EB were responsible for the design. HB and OSD did the data analysis. EB contributed to the interpretation of the results. HB was responsible for drafting the manuscript, and OSD and EB contributed to writing of the article. All three approved the final version of the article.
